# Ultralow Temperature Sintering of High-Performance Sm-Doped Pb(Zr,Ti)O_3_-Based Piezoelectric Ceramics

**DOI:** 10.3390/ma18030512

**Published:** 2025-01-23

**Authors:** Zechi Ma, Zixuan Yuan, Zhonghua Yao, Jiangxue Chen, Hua Hao, Minghe Cao, Hanxing Liu

**Affiliations:** 1 State Key Laboratory of Advanced Technology for Materials Synthesis and Processing, School of Material Science and Engineering, Wuhan University of Technology, Wuhan 430070, China; 2 Sanya Science and Education Innovation Park, Wuhan University of Technology, Sanya 572000, China; 3 International School of Material Science and Engineering, Wuhan University of Technology, Wuhan 430070, China

**Keywords:** piezoelectric ceramics, ceramics, morphotropic phase boundary, electromechanical response

## Abstract

Piezoelectric materials (PZTs) enjoy extensive applications in the field of electromechanical sensors due to their sensitive response to external electric fields. The limited piezoelectric response for single-layer piezoceramic pellets drives the use of multilayered technology to increase the electric displacement of a single piezo device. As is well known, Ag is commonly used as a metal for electrodes in devices based on traditional PZTs, which always densify at a high temperature above 1100 °C, resulting in Ag migration. Here, a high-performance samarium-ion-doped Sm_0.01_Pb_0.99_(Zr_0.54_Ti_0.46_)O_3_ ceramic was selected as parent materials to develop a new Ag-cofired ceramic matrix with a sintering temperature of 920 °C by glass flux. The ceramic composition with 2.0 wt% glass addition exhibits the excellent performance of piezoelectric *d*_33_~492 pC/N, planar electromechanical coupling coefficient *k*_p_~50.1%, mechanical quality factor *Q*_m_~68.71, and Curie temperature *T*_c_~356 °C, respectively. The cyclic stability of *d*_33_ was measured below 6.6% at 30 kV/cm, which indicates that the piezoceramic has good temperature stability and fatigue resistance. Therefore, this study provides a novel high-performance piezoelectric system to meet the cofired requirement for multilayered piezoelectric devices.

## 1. Introduction

Piezoelectric materials possess a critical electrical characteristic that can directly convert energy between mechanical energy and electrical energy. This capability finds applications in various fields, including biomedicine, consumer electronics, and defense technology. Typically, Pb(Zr,Ti)O_3_-based ceramics are widely taken as core materials in the manufacturing of piezoelectric devices due to their excellent electromechanical responses, such as sensors, transducers, electrostatic capacitors, and power sub-devices [1,2,3,4].

Element doping has been an effective way to elevate the performance of Pb(Zr,Ti)O_3_-based piezoelectric ceramics. There are three kinds of doping methods: donor doping, acceptor doping, and equivalent doping [5,6]. Among them, rare-earth elements are promising dopants used in PZT ceramics, such as La [7,8,9], Er [10,11,12], Gd [13,14], Eu [15,16], Nd [17,18]. Among these rare-earth doping, Sm has shown good modification effects in recent studies. For example, Li et al. [11] synthesized a Sm^3+^-modified Pb(Mg_1/3_Nb_2/3_)O_3_-PbTiO_3_ piezoceramic with a Curie temperature of 89 °C and found that the piezoelectric performance of the Sm-PMN-PT piezoelectric ceramic was significantly improved compared to as-known piezoelectric ceramics. Shruti et al. [19] developed a 2 at% Sm-doped PZT composition in the *T*_c_ region, achieving a large *d*_33_ of 915 pm/V. The exceptionally high piezoelectricity could be ascribed to Sm doping, which could induce a series of microscopic changes, such as increased internal stress, interphase boundary motion, and increased energy for domain wall movement. Guo et al. [20] prepared Sm-modified 0.4Pb(Mg_1/3_Nb_2/3_)O_3_-(0.6-x)PbZrO_3_-xPbTiO_3_ piezoceramics by introducing PbZrO_3_ with *T*_c_ = 232 °C to form a ternary system. At x = 0.352, Both *ε*_r_ of 4090, *T*_c_ of 184 °C, and *d*_33_ of 910 pC/N can be achieved due to synergistic effects of morphotropic phase boundary (MPB) and the increased local structural heterogeneity.

The creation of multilayer piezoelectric ceramic devices can fulfill the demands for miniaturization, reduce driving electric fields, and be cost-effective in products that utilize base metal electrodes. Commonly, Ag (melting point~961.8 °C) is selected as an internal electrode in multilayered piezoelectric devices [21]. So, the reduction in sintering temperature is needed for the piezoelectric matrix. Numerous attempts have been made, such as liquid phase additives [22,23], nanosized raw materials [24,25], and the cold sintering method [26,27]. A common method involves adding flux to create a liquid phase, which facilitates the dissolution and migration of materials and accelerates grain growth. A variety of additives can be selected as modifiers, such as B_2_O_3_ [28,29], CuO [30,31], Bi_2_O_3_ [8,32], ZnO [33,34], glass [35,36], and so on. For instance, Nan et al. [37] investigated the effects of lithium carbonate on the sintering behavior, microstructure, and functional properties of Ba_0.85_Ca_0.15_Zr_0.1_Ti_0.9_O_3_ piezoceramics by the sol–gel method. The results showed that the addition of 0.5 wt% Li_2_CO_3_ significantly could enhance the sintering density and reduce the sintering temperature near 1300 °C. Dai et al. [38] reported that adding 0.7 wt% CdCO_3_ could effectively reduce the sintering temperature of the 0.8Pb(Zr_0.48_Ti_0.52_)O_3_-0.125Pb(Zn_1/3_Nb_2/3_)O_3_-0.075Pb(Mn_1/3_Nb_2/3_)O_3_ ceramics from 1150 °C to 980 °C. A low sintering temperature of 940 °C was selected by Lin et al. [39] in PZT-based piezoelectric ceramics, and excellent piezoelectric performance could also be achieved with a lithium carbonate addition of 0.2 wt%, resulting in *k*_p_~66.7%, *Q*_m_~71, and *T*_c_~226 °C. Therefore, it is possible, in this case, that the addition of low-melting glass can reduce densifying temperature and maintain excellent piezoelectric properties.

In our previous work [40], Sm-modified composition, Sm_0.01_Pb_0.99_(Zr_0.54_Ti_0.46_)O_3_ exhibited promising electrotechnical responses with large ferroelectric polarization (*P*_r_ = 40.9 μC/cm^2^, *E*_c_ = 14.7 kV/cm), accompanying a piezoelectric *d*_33_ of 590 pC/N, a *k*_p_ of 57.1%, an electric strain S_max_ of 0.31%, a *d*_33_* of 632 pm/V, and an especially high *T*_c_ above 330 °C, respectively. Here, the aim of this project is to develop a low-sintered composition to cofire with Ag electrodes for the fabrication of multilayer piezoelectric actuators. However, this composition Sm_0.01_Pb_0.99_(Zr_0.54_Ti_0.46_)O_3_ exhibited a high densification temperature above 1200 °C, exceeding the melting point of silver. Furthermore, silver migration always happens in the ceramic–electrode interfaces if the cofiring temperature is above 950 °C [41]. In view of this, a commercial lead-free glass (LF-glass) flux with a low melting point of ~650 °C was chosen to reduce the cofiring temperature, and the target temperature is 920 °C to meet the cofiring requirements.

## 2. Materials and Methods

Raw materials including PbO (97% purity, Shanghai Aladdin Reagent Co., Ltd., Shanghai, China), TiO2 (99.9% purity, Shanghai Aladdin Reagent Co., Ltd., Shanghai, China), ZrO2 (99.99% purity, Shanghai Aladdin Reagent Co., Ltd., Shanghai, China), and Sm_2_O_3_ (99.9% purity, Shanghai Aladdin Reagent Co., Ltd., Shanghai, China) were used with analytically pure grade. LF-glass powder was selected as sintering aid. The LF-glass powders with an initial melting temperature of ~500 °C were bought from Anmi Anywhere New Materials (Guangzhou) Co., Ltd. (Guangzhou, China). The Sm_0.01_Pb_0.99_(Zr_0.54_Ti_0.46_)O_3_ (SPZTO) compositions were synthesized through solid-state reactions. The materials were mixed in stoichiometric ratios and milled for 4 h at room temperature, then calcined at 900 °C for 3 h and ball-milled at room temperature again. The resultant mixture was granulated with PVA solution and subsequently pressed into disk-shaped pellets (*Φ* = 12 mm, *d* = 1 mm) under the pressure of 300 MPa. The binder was burned out, and the pellets were sintered at 920 °C for 3 h.

X-ray diffraction (XRD) analysis was conducted using a PANalytical diffractometer (Almelo, The Netherlands) with Cu *k*_α_ radiation and a 2θ step size of 0.02°. Silver-plated ceramics were prepared for electrical measurement. The samples were poled in dimethyl silicone at 30 kV/cm and 120 °C for 20 min and piezoelectric properties were evaluated for the samples after one-day aging. An E4980A LCR meter (Santa Rosa, CA, USA) and a quasi-static piezoelectric *d*_33_ instrument (Type: ZJ-3A, Beijing, China) were used for dielectric temperature spectroscopy up to 360 °C at a heating rate of 1.5 °C/min. The S-E relationship was measured with a multilayer actuator system at 30 kV/cm and 10 Hz, while electromechanical coupling and piezoelectric coefficients were assessed using an HP4294 impedance analyzer (Santa Rosa, CA, USA). The *k*_p_ value and the mechanical quality factor *Q*_m_ were calculated according to Equations (1) and (2):

(1) $$k_{p}=\frac{1}{\sqrt{0.395\frac{f_{r}}{f_{a}-f_{r}}+0.574}}$$

(2) $$Q_{m}=\frac{{f_{a}}^{2}}{2\pi f_{r}R(C_{0}+C_{1})({f_{a}}^{2}-{f_{r}}^{2})}$$

where parameters *f*_r_ and *f*_a_ denote the resonance frequency and anti-resonance frequency; *R* denotes the minimum impedance at resonance; C_1_ and C_0_, respectively, represent capacitance of the ceramic and capacitance under static electric field at resonance.

## 3. Results and Discussion

**Figure 1:**
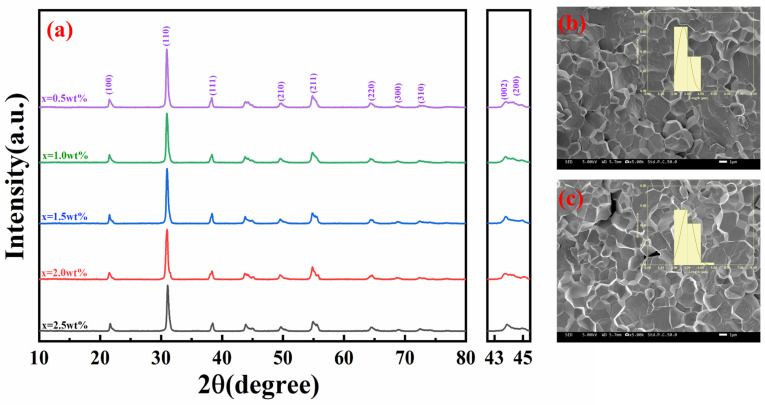
(**a**) XRD patterns of the SPZTO + x wt% LF-glass ceramics at room temperature; (**b**) SEM images of fracture cross of SPZTO + 0.5 wt% LF-glass ceramics sintered at 920 °C at room temperature; (**c**) SEM images of fracture cross of SPZTO + 2.0 wt% LF-glass ceramics sintered at 920 °C at room temperature.

Figure 1a presents the XRD patterns of SPZTO ceramics with varying LF-glass contents. The results indicate that a pure perovskite phase is achieved, confirming the development of a complete solid solution. The broadening of the (200) peak is observed in the samples at the 2θ = 44~46° region (in other words, near the morphotropic phase boundary (MPB)), which is evidence of the coexistence of the tetragonal and rhombohedral phases. It is generally believed that piezoelectric ceramics have the optimum piezoelectric properties near the MPB. When the LF-glass contents increase, a distinct splitting of the (200)/(002) peaks can be observed, which implies that the coexistence of the rhombohedral-tetragonal phase near MPB tends to transform into the tetragonal-rich phase, which may enhance piezoelectric properties by flattening the free energy profile. When the material’s planar free energy is higher, it means that the material can more easily undergo changes in spontaneous polarization under the influence of an external electric field, which directly enhances the piezoelectric effect. Typically, the addition of glass flux cannot modify the crystal structure of the SPZTO ceramics, indicative of the occupation of the glass phase at the grain boundary. Figure 1b,c depict scanning electron microscope (SEM) images of fractured surfaces after sintering at 920 °C with different LF-glass. It is observed that as the grain size of the ceramics is basically unchanged with increasing LF-glass, grain size has a significant impact on the electrical performance of ceramics. Preliminary analysis suggests that the ceramic samples show optimal electrical performances at x = 2.0. This finding is supported by the results from X-ray diffraction (XRD) spectroscopy, which provide additional confirmation.

**Figure 2:**
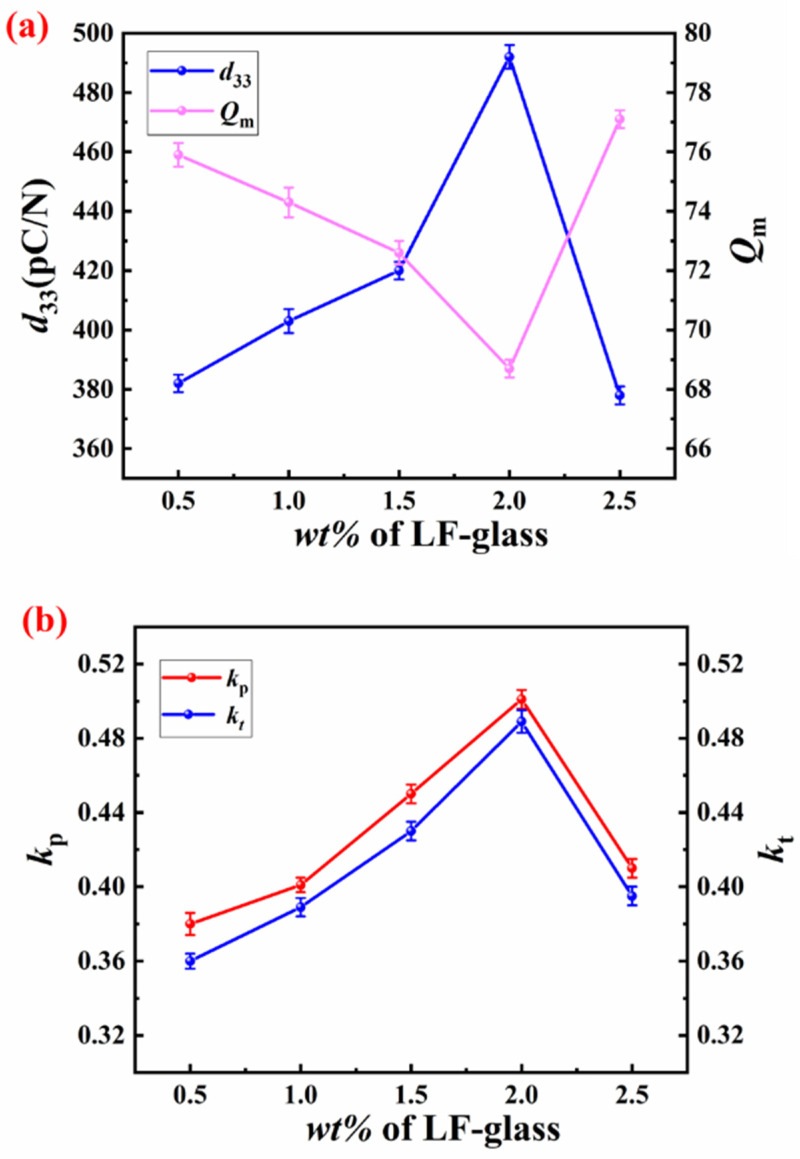
(**a**) The variation of *d*_33_ and *Q*_m_ at room temperature; (**b**) the variation of *k*_p_ and *k*_t_ of SPZTO + *x* wt% LF-glass ceramics at room temperature.

The piezoelectric coefficient quantifies the relationship between applied stress (or electric field) and the resulting electric displacement (or strain). Figure 2 shows piezoelectric performances of the glass-modulated SPZTO ceramics with different LF-glass contents. As shown in Figure 2, the enhanced piezoelectricity can be obtained at x = 2.0 with *d*_33_ = 492 pC/N, *Q*_m_ = 68.71, *k*_p_ = 50.1%, and *k*_t_ = 48.9%. This indicates that the SPZTO ceramic sintered at 920 °C can be effectively fabricated, facilitating cofiring with a Ag electrode to prepare a multilayer-structured piezoelectric harvester. At x = 2.5%, redundant LF-glass cannot be completely discharged, affecting the electrical properties of the material.

**Figure 3:**
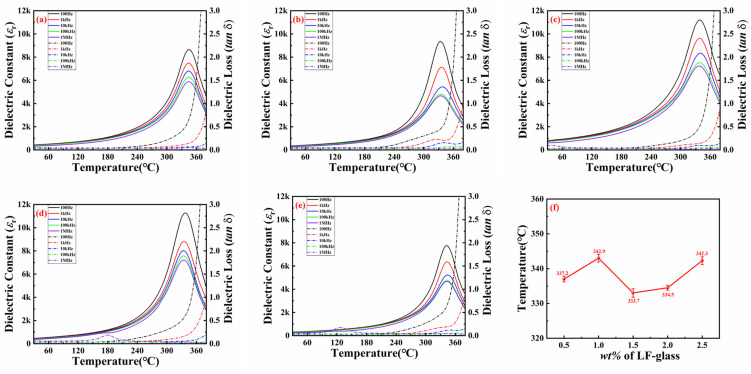
Dielectric–temperature spectra of the SPZTO + *x* wt% LF-glass ceramics: (**a**) *x* = 0.5; (**b**) *x* = 1.0; (**c**) *x* = 1.5; (**d**) *x* = 2.0; (**e**) *x* = 2.5; (**f**) the variation of *T*_c_ under different LF-glass content.

Figure 3 depicts dielectric property–temperature spectra of the SPZTO ceramics with various frequencies (100 Hz, 1 kHz, 10 kHz, and 100 kHz). As the testing frequency increases, there is no noticeable shift or broadening of the dielectric peak. This suggests the absence of relaxor behavior in the dielectric maximum peaks. With the increase in the LF-glass added, the *T*_c_ of the ceramic remains around 336 °C without any obvious change. Poled piezoelectric ceramics typically exhibit instability at temperatures slightly above half of the Curie temperature, which is suitable for their use as high-precision sensors. It can be inferred that piezoelectric devices made from these compositions are applicable at temperatures exceeding 160 °C.

It can be observed that abnormal dielectric loss peaks far below Tc can be observed in high doping compositions for x = 2.0 at 180 °C and for x = 2.5 at 120 °C. It is possible that the extra dielectric loss peaks far below *T*_c_ can be ascribed to the doping glass.

**Figure 4:**
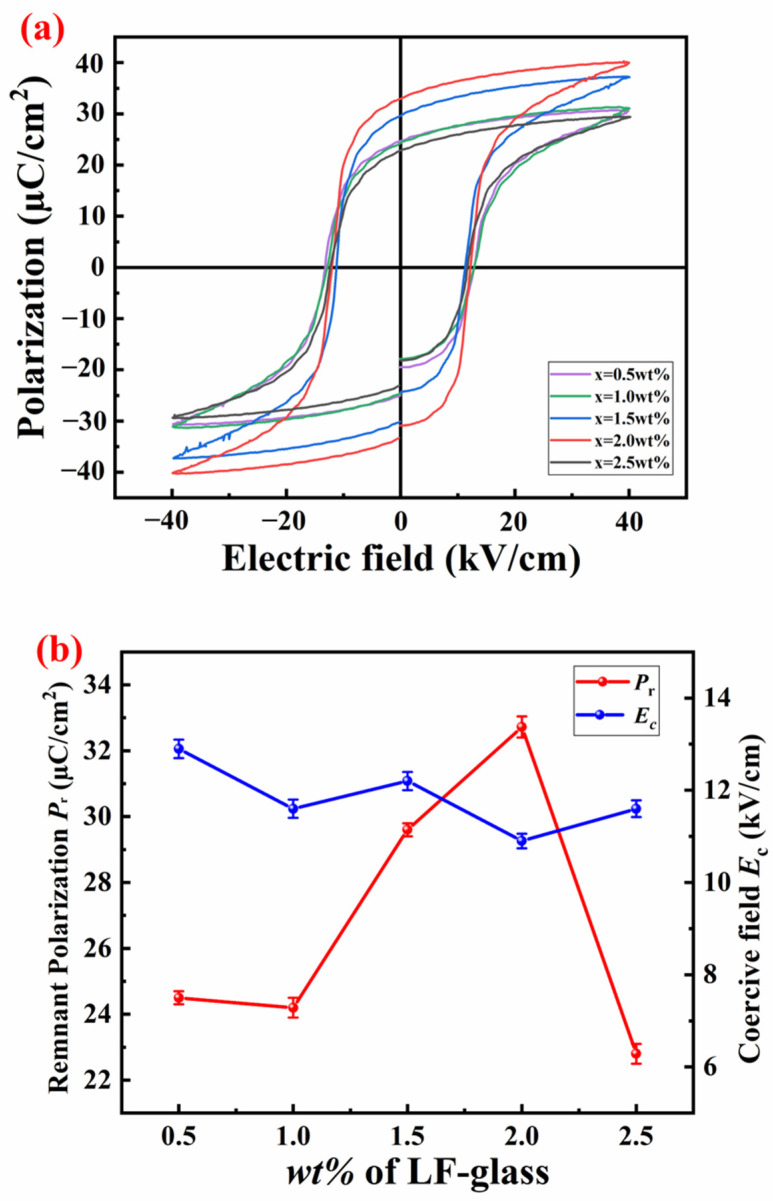
(**a**) *P*-*E* hysteresis loops of the SPZTO + *x* wt% LF-glass ceramics at room temperature; (**b**) the variation of *P*_r_ and *E*_c_ under different LF-glass content at room temperature.

Figure 4a shows the P-E hysteresis loop at room temperature and polarization characteristics of SPZTO ceramics. Saturation polarization tests were achieved on the studied ceramic without breakdown. Figure 4b illustrates the trends in remnant polarization *P*_r_ and coercive field *E*_c_ for each composition. The SEM test results show that the samples obtained at x = 1.5 and 2.0% have larger grain sizes. As the grain size increases, the remnant polarization *P*_r_ of the PZT ceramic gradually increases, while the coercive field *E*_c_ gradually decreases. It is commonly understood that a lower *P*_r_ or a higher *E*_c_ indicates greater resistance to domain reversal, which is likely attributed to the influence of grain boundaries that restrict domain switching in samples with smaller grain sizes. Notably, the ceramics at x = 2.0 demonstrate the highest remnant polarization of 32.72 µC/cm^2^, along with the lowest coercive field of 10.9 kV/cm. This indicates significant potential for enhanced piezoelectric activity in this specific composition.

**Figure 5:**
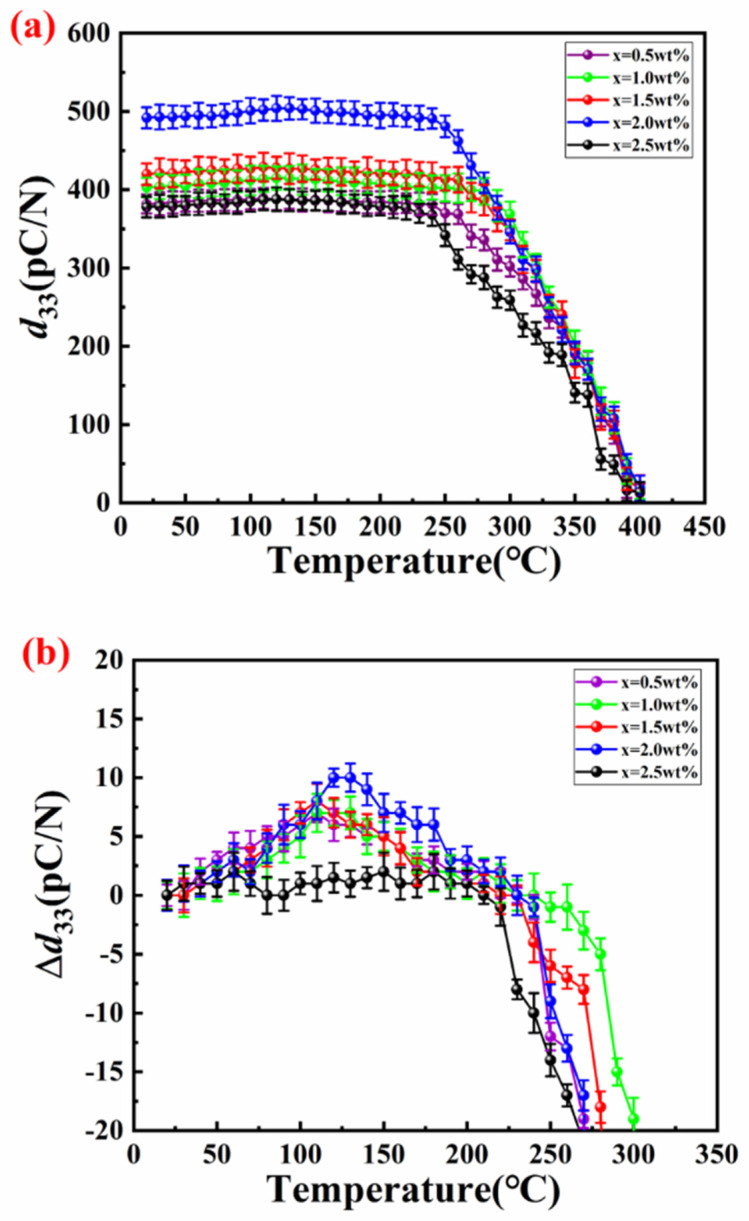
(**a**) The in situ *d*_33_–temperature dependence of the SPZTO at room temperature + *x* wt% LF-glass ceramics; (**b**) the Δ*d*_33_–temperature dependence of Sm_0.01_Pb_0.99_(Zr_0.54_Ti_0.46_)O_3_ + *x* wt% LF-glass ceramics at room temperature.

Typically, the operating temperature of piezoelectric ceramics is maintained below half of the Curie temperature to ensure optimal performance. This sensitivity to temperature is crucial, as depolarization can significantly affect the reliability and functionality of the device. Figure 5 shows the in situ *d*_33_ values of the piezoelectric ceramics exhibit variations with temperature. This figure illustrates how the piezoelectric response changes, highlighting the importance of temperature management in the application of these materials. The variation in *d*_33_ is less than 3%, indicating that *d*_33_ remains stable near *T*_c_, attributed to the lower Gibbs free energy in the polarization curve at the ceramic boundary [42]. Figure 5b shows a slight increase in *d*_33_ as the temperature rises. It is widely recognized that compositions near the morphotropic phase boundary (MPB) typically exhibit an active mixed-phase behavior [43].

**Figure 6:**
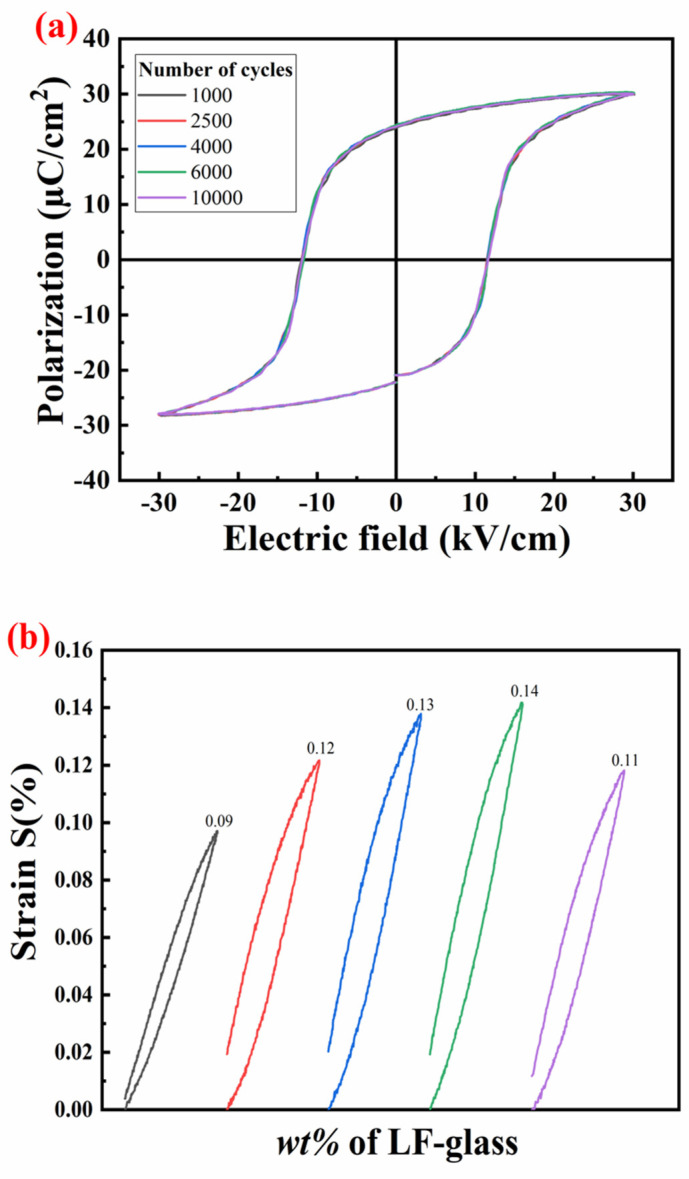
Cyclic and strain test diagrams of the SPZTO + *x* wt% LF-glass ceramics at room temperature; (**a**) the cyclic *P-E* curve of LF-glass = 2 wt% under 30 kV/cm; (**b**) *S-E* curves of ceramics under 30 kV/cm at room temperature.

The orientation and arrangement of ceramic dipoles may alter under the influence of stress or an electric field, thereby impacting the material’s piezoelectric properties. During multiple cycles of loading and unloading, the charge distribution and lattice structure of the ceramic material may change, affecting its piezoelectric properties. Furthermore, the DC electric field aligns most domains, resulting in internal stress and an increased concentration of defects within the ceramics. When a DC electric field is applied to ferroelectric ceramics, it tends to align the domains, but this process generates internal mechanical stresses and can lead to the formation of defects due to the strain from the domain reorientation [44]. Consequently, cyclic performance emerges as another critical factor in determining the piezoelectric, ferroelectric, and dielectric properties of the materials. Figure 6a presents the changes in the P-E curves of the ceramic sample at x = 2 before and after 10^4^ cycles under a 30 kV/cm electric field. Notably, after 10^4^ cycles, the variation rate remains below 6.6%, demonstrating strong fatigue resistance and exceptional stability and reliability for prolonged use. The ceramics experience field-induced strain in response to an external electric field due to the inverse piezoelectric effect and the irreversible motion of domain walls. Figure 6b illustrates the continuous relationship between strain and electric field for all samples. Different compositions display varying strain responses when subjected to electric fields at a driving voltage of 30 kV/cm. When x = 2, the ceramic reaches a strain of 0.14%, comparable to traditional PZTs, which exhibit around 0.1%. Furthermore, the strain curves of the components of piezoelectric ceramics show excellent linearity, meaning that they can generate corresponding deformations in a stable and predictable manner when the electric field changes. This linear response is the foundation of the performance of piezoelectric actuators, ensuring the stability, predictability, and efficiency of the actuator. Therefore, the excellent linear characteristics make piezoelectric ceramics an ideal driving material.

## 4. Conclusions

In this work, high-performance SPZTO ceramics were prepared. LF-glass was used as a flux, and the sintering temperature was reduced to 920 °C with LF-glass of 2.0 wt% to achieve the best performance, including *d*_33_~492 pC/N, *k*_p_~50.1%, *Q*_m_~68.71, *T*_c_~356 °C, and the ceramics could be fired with Ag in multilayer structure piezoelectric device fabrication. Under a voltage of 30 kV/cm, the piezoelectric strain is expected to reach 0.14%. Moreover, the ceramics maintained excellent stability and reliability over time and showed good temperature stability, indicating the potential of multilayer-structured piezoelectric devices.

## Data Availability

The original contributions presented in this study are included in the article. Further inquiries can be directed to the corresponding author.
